# An update of genetic basis of PCOS pathogenesis

**DOI:** 10.20945/2359-3997000000049

**Published:** 2018-05-07

**Authors:** Raiane P. Crespo, Tania A. S. S. Bachega, Berenice B. Mendonça, Larissa G. Gomes

**Affiliations:** 1 Universidade de São Paulo Hospital das Clínicas Faculdade de Medicina Universidade de São Paulo São Paulo SP Brasil Divisão de Endocrinologia e Laboratório de Hormônios e Genética Molecular (LIM-42), Hospital das Clínicas da Faculdade de Medicina da Universidade de São Paulo (HCFMUSP), São Paulo, SP, Brasil

**Keywords:** Polycystic ovary syndrome, PCOS, genetics, pathogenesis, complex genetic trait

## Abstract

Polycystic ovary syndrome (PCOS) is a common and complex endocrine disorder that affects 5-20% of reproductive age women. PCOS clinical symptoms include hirsutism, menstrual dysfunction, infertility, obesity and metabolic syndrome. There is a wide heterogeneity in clinical manifestations and metabolic complications. The pathogenesis of PCOS is not fully elucidated, but four aspects seem to contribute to the syndrome to different degrees: increased ovarian and/or adrenal androgen secretion, partial folliculogenesis arrest, insulin resistance and neuroendocrine axis dysfunction. A definitive etiology remains to be elucidated, but PCOS has a strong heritable component indicated by familial clustering and twin studies. Genome Wide Association Studies (GWAS) have identified several new risk loci and candidate genes for PCOS. Despite these findings, the association studies have explained less than 10% of heritability. Therefore, we could speculate that different phenotypes and subphenotypes are caused by rare private genetic variants. Modern genetic studies, such as whole exome and genome sequencing, will help to clarify the contribution of these rare genetic variants on different PCOS phenotypes. Arch Endocrinol Metab. 2018;62(3):352-61

## INTRODUCTION

Polycystic ovary syndrome (PCOS) is a common and complex endocrine disorder that affects 5 to 20% of reproductive age women, and it is a major cause of hirsutism and anovulatory infertility (1). However, PCOS is more than a reproductive disease; it is associated with a wide range of metabolic disorders, such as glucose intolerance, diabetes, dyslipidemia, hypertension, hepatic steatosis, and increased cardiovascular surrogate markers. An increased prevalence of cardiovascular outcomes and mortality has not yet been demonstrated in this population (2).

PCOS diagnosis is currently based on a combination of the following signs and symptoms: hyperandrogenism and/or hyperandrogenemia, ovulatory dysfunction and polycystic ovarian morphology (PCOM). In the first attempt to define diagnostic criteria, the National Institutes of Health (NIH) Conference proposed in 1990 that the presence of both hyperandrogenism and chronic anovulation were mandatory for diagnosis (3) (Table 1). In 2003, the Rotterdam Consensus included PCOM finding as a criterion, and defined that the presence of at least two out of the three main characteristics were necessary to confirm the diagnosis (Table 1) (4). Therefore, the Rotterdam Consensus expanded the possibilities of combinations of the three classic manifestations, allowing the characterization of four main phenotypes (Table 2). In 2006, the Androgen Excess Society proposed a modification, in which hyperandrogenism would be an essential condition for diagnosis, reducing the number of phenotypic possibilities (5). More recently, a Consensus by NIH tried to unify the recommendations, suggesting the use of the Rotterdam Consensus for PCOS diagnosis in order to include the four phenotypes possibilities, and thus expanding the population with PCOS to its largest number (6).

**Table 1:** Diagnostic criteria for PCOS

NIH 1990	Rotterdam 2003	AE-PCOS Society 2006
•Chronic anovulation •Clinical and/or biochemical signs of hyperandrogenism (*Both criteria needed*)	•Oligo- and/or anovulation •Clinical and/or biochemical signs of hyperandrogenism •Polycystic ovaries (*Two of three criteria needed*)	•Clinical and/or biochemical signs of hyperandrogenism •Ovarian dysfunction (Oligo-anovulation and/or polycystic ovarian morphology) (*Both criteria needed*)

**Table 2:** PCOS phenotypes according to each diagnostic criteria

Phenotypes	1	2	3	4

	Classic	NIH	Ovulatory	Normoandrogenic
Hyperandrogenism	Yes	Yes	Yes	No
Chronic anovulation	Yes	Yes	No	Yes
Polycystic ovaries	Yes	No	Yes	Yes
NIH 1990	X	X		
Rotterdam 2003	X	X	X	X
AE-PCOS Society 2006	X	X	X	

## CLINICAL ASPECTS: WIDE HETEROGENEITY

There is wide clinical heterogeneity, in terms of not only the main components of the syndrome, but also of the metabolic aspects. Cross-sectional studies have shown that metabolic abnormalities are more common in the classic and NIH phenotypes, often classified together with the denomination of classic (also adopted in this current review), compared to the other Rotterdam phenotypes, namely, ovulatory and normoandrogenic (Table 2). Moghetti and cols. found that the prevalence of each phenotype is about 70% for classic, 15% for ovulatory and 15% for normoandrogenic phenotypes (7). Other epidemiological studies in unselected populations reported the prevalence of 40–45% for classic phenotype, ~35% for ovulatory phenotype and ~20% for normoandrogenic phenotype (8). The prevalence of insulin resistance in PCOS patients is around 70%; however, insulin resistance differs among phenotypes: 80% in the classic, 65% in the ovulatory and only 38% in the normoandrogenic phenotype (7). The presence of metabolic syndrome also differs among these groups. Çelik and cols. compared 183 normoandrogenic PCOS patients with 504 classic/ovulatory PCOS patients and found a significantly higher prevalence of metabolic syndrome (OR 2.95) in the classic/ovulatory group (9). This clinical and metabolic heterogeneity could be explained by distinct pathogenic causes.

## PCOS PATHOGENESIS ASPECTS

### Ovarian and adrenal hyperandrogenism

**Figure 1:**
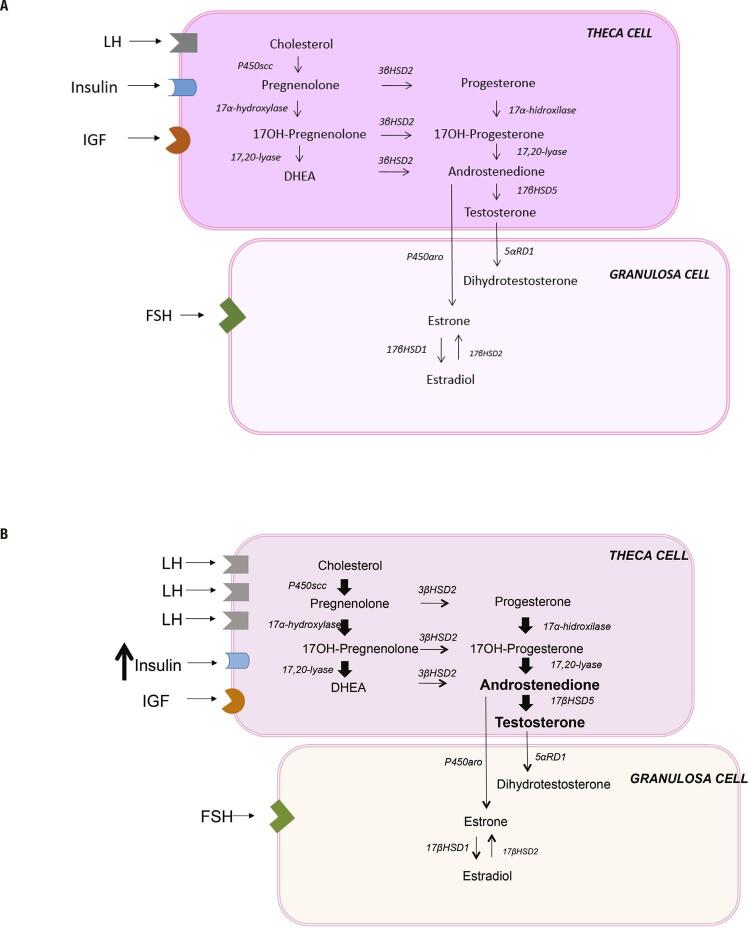
Ovarian steroidogenesis. (A) Normal ovarian steroid synthesis. (B) PCOS ovarian steroid synthesis. In comparison to normal theca cells, PCOS theca cells show increased expression of LH receptor and increased expression of CYP17A1 gene, leading to enhance of 17α-hydroxylase and 17,20-lyase activity, and amplifying androgen synthesis. Exogenous factors, such as hyperinsulinemia and IGFs are modulatory hormones that can disrupt normal intra-ovarian regulatation of steroidogenesis.

There are considerable evidences, an that PCOS is an intrinsic disorder of the ovaries and that the primary defect resides in the increased androgen biosynthesis. Normally, ovarian theca cells produce androgens in response to LH. The theca cells express the *CYP17A1* gene, which encodes the P450c17 enzyme, catalyzing both 17α-hydroxylase and 17,20-lyase activity, the rate-limiting step in sex steroid synthesis. Androgen production is cyclical and modulated by intra-ovarian and extra-ovarian mechanisms. As LH rises, a downregulation of LH receptors and a decrease in CYP17A1 expression occur, minimizing the androgenic production. Estrogen and androgen inhibit 17α-hydroxylase and 17,20-lyase activity in a paracrine and autocrine negative feedback loop. In contrast, insulin and IGFs stimulate the P450c17 enzyme and up-regulate LH receptor sites (10) (Figure 1).

PCOS ovaries are typically hypersensitive to LH, which is caused by partial escape from LH receptor downregulation. The imbalance of the intra-ovarian regulatory system seems to play a role in this increased sensitivity to LH. Exogenous factors, such as insulin resistance and hyperinsulinemia, are examples of extra-ovarian modulators that disrupt normal intra-ovarian regulatory mechanisms (10) (Figure 1).

In vitro studies have demonstrated that isolated theca cells or tissues from PCOS women secrete more androgen than cells from normal controls. The increased androgen synthesis in PCOS theca cells results from increased expression of 17α-hydroxylase/17,20-lyase and the cholesterol side-chain cleavage enzyme, suggesting an intrinsic theca cell defect (11). Taken together, these data indicate that ovarian cells from PCOS patients have intrinsic and stable characteristics that contribute to their phenotype, even after exclusion of gonadotrophin, hyperinsulinemia and hyperandrogenism stimulus.

Although the ovaries are considered the main source of androgens in PCOS, increased adrenal androgens, mainly DHEA and DHEAS, are present in around 20–30% of PCOS women (12). Normal adrenal *zona reticularis* steroidogenic enzymes resemble theca cell enzymes, favoring the formation of DHEA, which is rapidly sulfated to DHEAS by sulfotransferase 2A1 (SULT2A1). DHEAS is the most abundant adrenal precursor, but it is an inert terminal product. However, DHEAS can be converted to DHEA, which can be metabolized into more potent androgens. Genetic variants in *SULT2A1* have been shown to alter the DHEAS/DHEA ratio in PCOS (13).

Increased adrenal P450c17 activity is also suggested in PCOS women with adrenal hyperandrogenism. This adrenal hyperandrogenism does not seem to depend on an increased hypothalamic–pituitary-adrenal drive, but it reflects a generalized adrenal hyper-responsiveness for androgenic biosynthesis (12).

Genetic and epigenetic factors are probably implicated in the theca cell and adrenal steroidogenesis dysfunction. An example of a genetic cause of adrenal PCOS is cortisone reductase deficiency. The original study described mutations in 11β-hydroxysteroid dehydrogenase (11βHSD) type I and hexose-6-phosphate dehydrogenase in a digenic triallelic mode of inheritance. In11β-HSD type I deficiency, cortisone is not convert into cortisol, with a consequent elevation of ACTH, which stimulates androgen production, leading to the PCOS phenotype (14).

### Ovarian folliculogenesis

The beginning of follicular recruitment is an intrinsic ovarian process, independent of gonadotropins. The primordial follicles are maintained in the quiescence state by epithelial-mesenchymal interactions, inhibitory transcription factors (LKB1/STK11/ BMP4) and pro-apoptotic factors (FOX) secreted by oocytes. A set of local follicular factors regulates follicular recruitment (GDF9, BMP6 and BMP9) (10), follicle growth and development. For example, BMP15 acts synergistically with GDF9 proliferating the granulosa cells (10).

**Figure 2:**
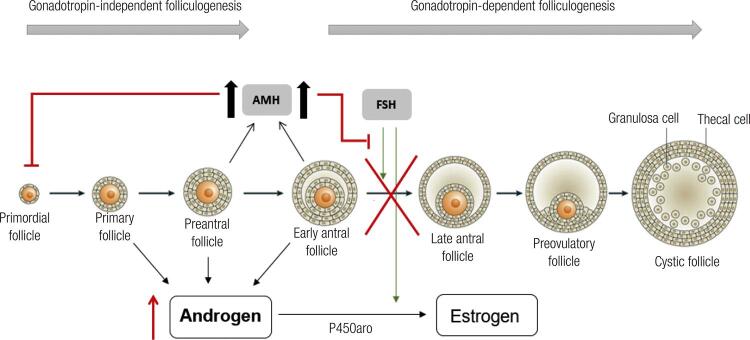
Role of AMH in folliculogenesis. AMH has an inibitory effect on initial recruitment of primary follicles, on FSH-dependent follicular maturation and selection of dominant follicle, and on FSH-induced aromatase expression on granulosa cells, reducing the conversion of testosterone into estradiol. Higher AMH levels in PCOS patients turns the follicles more resistant to FSH action, culminating in inhibition of follicular maturarion and ovulation, and in inhibiton of aromatase expression, and consequently, leading to hyperandrogenism. Adapted from Azziz and cols. 2016 (1).

Another important modulator of folliculogenesis is AMH, a member of the GFR-beta superfamily that is produced by the granulosa cells of small growing follicles. The highest expression of AMH is detected in preantral and small antral follicles of ≤ 4 mm. Once the follicles reach > 8 mm, AMH expression is reduced, and these follicles become more sensitive to FSH action, culminating with increased follicle growth and estrogen production, selection of the dominant follicle and subsequent ovulation in the normal ovary (15). AMH acts to prevent the initial FSH-independent recruitment of primary follicles from the primordial follicle pools, inhibits FSH-dependent follicular maturation and the selection of the dominant follicle (1) (Figure 2).

Several studies have shown that serum AMH levels are significantly higher in patients with PCOS (15-17), and this finding is compatible with the large number of follicles in the preantral and antral stages, both of which are stages of maximum AMH production. Pellatt and cols. demonstrated that the production of AMH per cell is 75 times greater in the granulosa cells of anovulatory PCOS patients (16). This finding suggests that high serum AMH levels are not only a consequence of the increased granulosa cell mass in anovulatory patients, but are also a potential causal factor for anovulation in PCOS (15,16). However, the cause for the increased production of AMH per granulosa cell remains unknown. Several other signals produced by granulosa cells and oocytes modulate (positively and negatively) the ovarian environment for oocyte maturation. These factors include inhibin B, TGF-beta superfamily members (ex. BMPs), cytokines (ex. TNF-alpha), and microRNAs (10).

Regulatory folliculogenesis factors, such as GDF9 and BMP15, have already been studied as possible causes of PCOS, but there is still no evidence that these molecules are responsible for abnormal folliculogenesis (18). Mutations of *BMP15* have been implicated in early ovarian failure caused by ovarian dysgenesis (19).

FOX transcription factors have also demonstrated a potential role in PCOS pathogenesis. Mikaeili and cols. correlated FOX03 expression and activation in granulosa cells with apoptosis rates, and found a positive correlation between them, making this transcription factor a potential candidate target (20).

### Insulin resistance

In 1970, rare forms of severe insulin resistance (IR), *acanthosis nigricans* and hyperandrogenism, were described (Insulin Resistance type A, Rabson-Mendenhall Syndrome and Leprechaunism) (21). The molecular mechanism of insulin resistance in these diseases was explained by a reduction in the binding of insulin to its receptor and by a defect in insulin receptor autophosphorylation (22,23). Family forms of lipodystrophy with severe IR were also associated with hyperandrogenism at the same time (24). Remarkable hyperinsulinemia, as a common feature of these syndromes, suggested for the first time that insulin could directly stimulate androgen production (24).

In 1980, Burghen and cols. reported that women with PCOS had increased insulin levels in response to the oral glucose tolerance test that were not justified only by obesity (25). In addition, women with classical PCOS had acanthosis nigricans, which gave them a phenotypic picture similar to the rare IR syndromes described previously (25).

Observational studies suggest that in obese PCOS women, metabolic abnormalities related to IR and obesity have a greater impact on anovulation mechanisms than excess androgens (26). However, IR in PCOS patients is not necessarily associated with obesity. Some lean patients with PCOS present with IR, and even obese women have IR disproportionate to the degree of adiposity (27). Therefore, insulin resistance in PCOS is an intrinsic characteristic aggravated by obesity and not simply a consequence of obesity.

In PCOS syndrome, IR is not generalized in all tissues. Defects in metabolic actions of insulin occur in muscle and adipose tissues, but mitogenic and steroidogenic actions of insulin in ovaries are preserved (28). By acting on the ovarian insulin receptor, insulin amplifies the response of theca cells to LH and increases the expression of P450c17 and 3β-HSD2, leading to increased androgen production (29). This pathway is well illustrated by studies in rats presenting IR induced by an obesogenic diet; in these studies, the knock-out of insulin receptors improved hyperandrogenism and anovulation (30). Reaffirmation of the role of insulin in steroidogenesis is observed in the treatment of PCOS. All treatments that reduce serum insulin levels, whether weight loss due to lifestyle changes, bariatric surgery, metformin or thiazolidinedione, significantly diminish anovulation and hyperandrogenemia (31,32).

### Neuroendocrine axis

Since ovarian steroidogenesis is directly related to the gonadotropic stimulus, alterations of LH are indicated as one of the factors involved in the development of PCOS.

Progesterone is the primary regulator of GnRH pulsatility. It is known that PCOS patients present a GnRH-generating pulse resistance to negative feedback by progesterone, which results in a higher LH pulses frequency and/or amplitude (33). This phenomenon has already been attributed to hyperandrogenemia in several studies, in which treatment with flutamide for 4 weeks restored hypothalamic sensitivity to ovarian steroids (34). At this point, alterations of the gonadotropic axis appear to be secondary to hyperandrogenemia. Moreover, LH excess is an inconstant feature of PCOS, thus, it is difficult to attribute a primary role to LH excess in the pathogenesis of all PCOS patients. However, in patients with congenital virilization, changes in LH pulsatility appear to be determinant for the PCOS phenotype (10).

**Figure 3:**
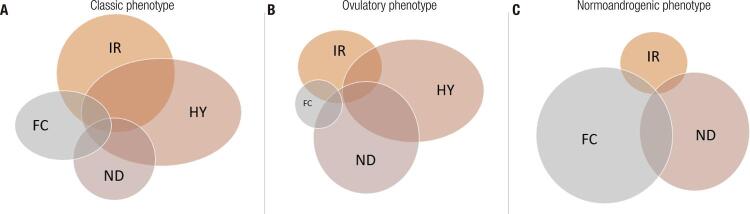
Schematic components of PCOS pathogenesis. Four main physiologic mechanisms contribute to PCOS pathogenesis: hyperandrogenism (HY), insulin resistance (IR), folliculogenesis dysfunction (FC), and neuroendocrine axis dysfunction (ND). These mechanisms contribute to each phenotype in different degrees. The classical phenotype shows alterations in all four mechanism, being HY and IR the main ones (A). In the ovulatory phenotype, HY and ND predominate and folliculogenesis seems not compromised (B). In contrast, in the normoandrogenic phenotype, FC seems to play the most important role in the pathogenesis, while HY is not present (C).

Therefore, ovarian and adrenal hyperandrogenism, altered folliculogenesis, increased insulin resistance and neuroendocrine axis dysfunction could have a greater or lesser role in PCOS pathogenesis (Figure 3). The classification of specific clinical patterns in patients with PCOS could improve the search for genetic determinants and potentially the development of specific treatment regimens.

## PCOS GENETIC HERITABILITY

Since 1968, studies have suggested an important genetic role contributing to the etiology of PCOS (35). First-degree relatives of PCOS patients have a higher risk of being affected by the syndrome compared to the general population. Kahsar-Miller and cols. studied first-degree relatives of 93 PCOS patients and found that 35% of non-menopausal mothers and 40% of sisters were also affected (36).

Examining a large twin cohort of 1332 monozygotic and 1873 dizygotic twin sisters, Vink and cols. found a higher correlation for PCOS in monozygotic compared to dizygotic twins. This study concluded that the genetic component contributes to over 70% of PCOS pathogenesis (37). Therefore, PCOS is a complex genetic disease with high inheritance rates and heterogeneous phenotypes.

Studies based on candidate genes for PCOS have not been successful. Studies involving more than 100 candidate genes, especially those related to reproductive axis, insulin resistance and chronic inflammation have not shown reproducible results (38,39). The lack of consistency across studies may stem from the small sample size, phenotypic heterogeneity among patients, an inadequate control group, the lack of comorbidity matching (obesity, insulin resistance), or the limitation to only one or two variants genotyped in each gene of interest instead of the whole gene (40).

The genome-wide association study (GWAS) is a more recent genomic approach to understanding the genetics of complex diseases. This method searches the genome for single nucleotide variants that occur more frequently in people with a particular disease than in people without the disease. The goal is to identify genetic risk factors for common diseases. Two GWAS conducted in Han Chinese women identified 11 loci, accounting for 17 SNPs, with a strong association risk for PCOS (41,42) (Table 3). These data were validated in Caucasians, confirming the presence of 12 out of the 17 variants with the same risk effect, indicating a similar genetic risk profile in different populations (43-48). However, the frequency of risk alleles differs significantly among populations (43).

**Table 3:** PCOSGWAS susceptibility gene markers

Han Chinese: 2011 (41), 2012 (42)	European 1 2015 (47)	European 2 2015 (48)
*LHCGR*	*GATA4/NEIL2*	*ERBB4*
*FSHR*	*FSHB*	*FSHB*
*C9orf3*	*C9orf3*	*RAD50*
*THADA*		*THADA*
*DENND1A*		*KRR1*
*YAP1*		*YAP1*
*RAB5B5*		
*HMGA2*		
*TOX3*		
*INSR*		

GWAS results have provided new insights into the biological pathways that may be involved in PCOS pathogenesis. The *DENND1A* (Differentially Expressed in Normal and Neoplastic Development isoform A1) gene was identified as a potential risk marker. *DENND1A* encodes a protein (connecdenn 1) associated with clathrin-coated pits where cell-surface receptors reside. The DENND1A protein is located in the cytoplasm and in the nuclei of theca cells (10). This gene has two major transcripts: DENND1A variant 1 (DENND1A.V1), which encodes a 1,009-aa protein with a C-terminal proline-rich domain, and DENND1A variant 2 (DENND1A.V2), which encodes a truncated 559-aa protein that contains the DENN domain and a clathrin-binding domain; however, the protein lacks the proline-rich domain and includes a C-terminal 33-aa sequence that is not found in the larger connecdenn variant 1 (49). McAllister’s group showed that DENND1A.V2 protein is more expressed in PCOS theca cells compared to normal theca cells. They also forced overexpression of DENND1A.V2 in normal theca cells, resulting in increased CYP17A1 and CYP11A1 expression and increased androgen biosynthes, compatible with PCOS theca cells profile. Additionally, knock-out of DENND1A.V2 in PCOS theca cells reduced CYP17A1 and CYP11A1 expression and androgen biosynthesis (10,49). Recently, DENND1A expression in the adrenal *zona reticularis* was shown (50). Taking all these data into consideration, the DENND1A variant 2 is potentially one of the mechanisms involved with the intrinsic abnormality in ovarian theca cells steroidogenesis in PCOS.

Some other genes (*LHCGR, FSHR, INRS, THADA*, *HMGA2, RAB5B*, *SUOX, YAP*, *ZNF217*) located in susceptibility loci indicated by GWAS studies were related to the gonadotropic regulatory axis, glucose and lipid metabolism, and cell cycle regulation.

- The *LHCGR* (luteinizing hormone/choriogonadotropin receptor) gene is a G protein-coupled receptor, which is expressed in granulosa cells, mainly in the preovulatory follicles. Increased LHCGR expression in granulosa cells in preovulatory follicles allows these follicles to respond to the LH peak in the middle of the cycle, resulting in ovulation. Inactivating mutations of this gene result in increased LH levels, amenorrhea or oligomenorrhea and infertility (51,52). In contrast, activating mutations of this gene are associated with hyperandrogenism (53).
- The *FSHR* (follicle stimulating hormone receptor) gene is related to the ovarian response to FSH and appears to be a highly plausible candidate gene for PCOS. Inactivating mutations of FSHR lead to hypergonadotropic hypogonadism and follicles stagnation in the preantral stage (50). Polymorphic variations of this gene in patients with PCOS have been associated with increased FSH concentrations (44) and resistance to exogenous ovarian stimulation with gonadotrophins and clomiphene (54).
- The *INRS* gene is the most prominent gene associated with insulin resistance indicated by GWAS. Mutations in the tyrosine kinase domain of the insulin receptor are known causes of insulin resistance and severe hyperinsulinemia (55). Polymorphic variants in different exons have been reported as risk factors for PCOS, but the results were generally not consistent in subsequent studies (50).
- *THADA* and *HMGA2* genes have also been described in the GWAS studies involving patients with DM2 (56). *RAB5B* and *SUOX* genes are located at the susceptibility locus for DM1 (57). *YAP* and *ZNF217* genes do not have a known ovarian function but they are related to cell proliferation and apoptosis (58).

The wide genetic possibilities indicated by GWAS could be related to the phenotypic heterogeneity. However, these loci identified in GWAS so far explain less than 10% of PCOS heritability. The rationale for GWAS large-scale sequencing studies is “common disease is associated with common variants”, postulating that common diseases are related to allelic variants present in more than 1-5% of the population. From this principle, GWAS studies allow the identification of an enormous number of genetic variants associated with complex diseases; however, most of the variants individually or in combination confer small increments in risk (1.1-1.5 fold). Variants with lower frequency and larger effect size that are not captured by GWAS may account for the deficit in heritability found using this method (59).

Moreover, the associations of the PCOS risk variants and some PCOS features, such as testosterone levels, hyperinsulinemia, and ovarian morphology could not be clearly established. The next step in genomics era is understanding how these variants contribute to disease’s pathogenesis as discussed by Jones and Goodarzi (60).

## GENETIC APPROACH IN PCOS POS-GWAS DISCOVERIES

Currently, GWAS have provided insights into the genetics of PCOS, pointing to some susceptible *loci*, usually in a non-coding region associated with the disease, but at the same time, these studies have shown that this complex disease cannot be explained by a limited number of variants with a low effect on phenotype. Much of the speculation about missing heritability from PCOS GWAS studies has focused on the role of rare genetic variants with substantial effect on phenotype.

It is in this context that massive parallel sequencing, which can process millions of sequences in parallel, provides a mechanism to screen the whole exome or to sequence the whole genome for rare variants. Whole exome sequencing (WES) has been able to advance the understanding of complex diseases via two approaches: searching for lower frequency coding variants that may only be present in subsets of the population and sequencing individuals with a severe phenotype. Rare variants with large effects in a specific gene may be found in extreme phenotypes, which can provide insights into the underlying pathophysiology of the common disorder (61).

Recently, Gorsic and cols. identified 18 rare variants in PCOS patients using WES, and 17 of 18 (94%) of the variants were associated with reduced AMH signaling. They hypothesize that these AMH mutations lead to the PCOS phenotype by disrupting AMH’s transcriptional inhibition of *CYP17A1*, leading to increased androgen biosynthesis (62). As demonstrated, WES seems promising in identifying rare genetic variants that contribute to PCOS pathogenesis and in mapping genetic variants that contribute to different aspects of each phenotype of the syndrome.

In conclusion, major advances in the genetics knowledge, combining with functional data and epigenetic studies in PCOS might increase our understanding of the etiology of this disease, with possible translational implications. The determination of genetic markers may improve the diagnosis of the syndrome and its phenotypes, allowing early intervention in associated co-morbidities and adequating treatment in a more individualized way.
